# Human Adenovirus Infection Causing Hyperinflammatory Syndrome Mimicking Multisystem Inflammatory Syndrome in Children (MIS-C): A Case Report

**DOI:** 10.7759/cureus.40239

**Published:** 2023-06-10

**Authors:** Kumar Diwakar, Tapas Sarangi, Preeti Srivastava, Sanjay K Tanti, Shikha Swaroop

**Affiliations:** 1 Pediatrics, Tata Main Hospital, Jamshedpur, IND; 2 Pediatrics, Manipal Tata Medical College, Jamshedpur, IND; 3 Pediatrics, Tata Steel Hospital, Noamundi, IND

**Keywords:** sars-cov-2, kawasaki disease, multisystem inflammatory syndrome in children, hyperinflammatory syndrome, human adenovirus infection

## Abstract

Transmission of human adenovirus (HAdV) infection and the associated clinical disease can be sporadic or epidemic and manifestations may range from mild infection to severe disease. HAdV has been seen to behave as a proinflammatory virus that can trigger the release of high levels of inflammatory cytokines and chemokines in children. Here, we report an unusual case of an infant with HAdV infection who presented with respiratory illness, with a protracted course, complicated with hyperinflammation and multi-system involvement with clinical characteristics mimicking multisystem inflammatory syndrome in children (MIS-C) and Kawasaki disease. The patient was an 11-month-old male infant with a background of infantile epilepsy, epileptic encephalopathy, hemimegaloencephaly, and global developmental delay, diagnosed as Ohtahara syndrome. He was admitted with a three-day history of cough, cold, fever, and respiratory distress. Management was initiated with a heated humidified high-flow nasal cannula and given ceftriaxone and hypertonic saline nebulization. Additionally, he developed loose motion on the fifth day of admission. The reverse transcriptase polymerase chain reaction (RT-PCR) of the nasopharyngeal swab was positive for HAdV. Due to persistent fever, elevated inflammatory markers, multisystem involvement (diarrhea, coagulopathy), an absence of a clear microbial etiology, and an epidemiologic link to severe acute respiratory syndrome coronavirus 2 (SARS-CoV-2) infection, MIS-C was diagnosed. The first dose of intravenous immunoglobulins (IVIG) was administered over the course of 48 hours and the baby required a second dose of IVIG as the fever failed to settle after the first dose. Within 24 hours of the second IVIG dose, defervescence occurred. His platelet count started to rise, and the baby developed thrombocytosis in the third week of illness. Echocardiography was suggestive of dilatation of mild left main coronary artery. He was weaned off oxygen support by day 14 and discharged on day 17. To our knowledge, this is the first reported case of HAdV infection with hyperinflammatory syndrome and vasculitis akin to MIS-C and Kawasaki disease.

## Introduction

Transmission of human adenovirus (HAdV) infection and the associated spectrum of clinical disease can be sporadic or epidemic. HAdV infection can manifest in a variety of ways, ranging from mild infection to severe disease, depending on the immunological state of the patient. Most cases in immune-competent hosts are self-limited, and fatalities are quite uncommon. HAdVs may cause a variety of cold-like symptoms, such as fever, cough, sore throat, and rhinorrhoea. Bronchitis, bronchiolitis, and pneumonia are typical lower respiratory diseases that can be severe and even fatal. HAdV infection can also be implicated in conditions like conjunctivitis, diarrhea, cystitis, myocarditis, cardiomyopathy, and meningoencephalitis.

Estimates of the prevalence of HAdV infection have been determined from serologic studies conducted in the 1960s, and those studies demonstrated that antibodies to strain HAdV-1, HAdV-2, and HAdV-5 are the most frequent and are found in 40-60% of children. Children between the ages of six months and five years are the ones who are most likely to contract HAdV. By the age of five, 50% of kids have antibodies to HAdV-5, and 70-80% of kids had neutralizing antibodies to HAdV-1 and HAdV-2. Antibodies against HAdV-3, HAdV-4, and HAdV-7 are infrequent in the same age ranges [1].

The term "cytokine storm" (CS) refers to a group of immune dysregulation disorders characterized by systemic inflammation, and multiorgan dysfunction that, if left untreated, can result in multiorgan failure. Various medications, infections, malignancies, autoimmune diseases, and monogenic disorders can all cause CS, a potentially fatal systemic inflammatory syndrome characterized by high levels of circulating cytokines and immune cell hyperactivation. CS has been shown to have a direct correlation with acute lung injury and the development of acute respiratory distress syndrome (ARDS) during viral infections including influenza viruses and coronaviruses [2]. HAdV has also been shown as a proinflammatory virus that can trigger the release of high levels of inflammatory cytokines and chemokines in children; the expression levels differ based on disease severity [3].

Hyperinflammatory syndrome in severe acute respiratory syndrome coronavirus 2 (SARS-CoV-2) is described as multisystem inflammatory syndrome in children (MIS-C). It was first reported in the United Kingdom in a cluster of eight children with SARS-CoV-2 infection manifesting as a hyperinflammatory syndrome with multiorgan involvement [4].

Here, we describe an unusual case of an infant with HAdV infection who presented with respiratory illness and progressed to develop hyperinflammation with multi-system involvement, manifesting with clinical characteristics that were like MIS-C and Kawasaki disease.

## Case presentation

**Figure 1:**
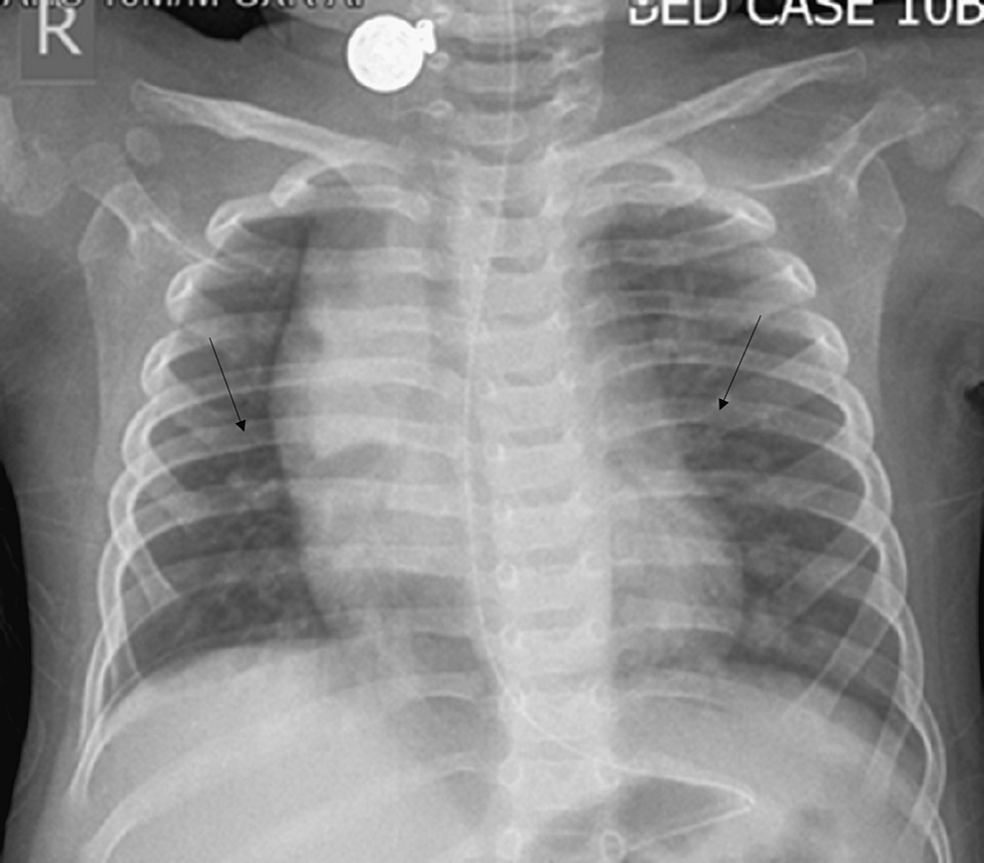
Prominent bronchovascular markings in a perihilar distribution.

The patient was an 11-month-old male infant with a background of infantile epilepsy, epileptic encephalopathy, hemimegaloencephaly, and global developmental delay, diagnosed as Ohtahara syndrome at two months of life. He was on multiple anti-epileptic drugs (AEDs) namely valproate, levetiracetam, clobazam, topiramate, and lacosamide. The child had a three-day history of cough, cold, and fever when he was seen and was admitted on account of respiratory distress. He had subcostal and intercostal muscle retractions, tachypnoea, and auscultation revealed crepitations and wheezing. He was febrile and his body temperature was 100.40F. He was provisionally diagnosed as a case of bronchiolitis and a chest X-ray (Figure 1) on admission, was consistent with bronchiolitis.

**Figure 2:**
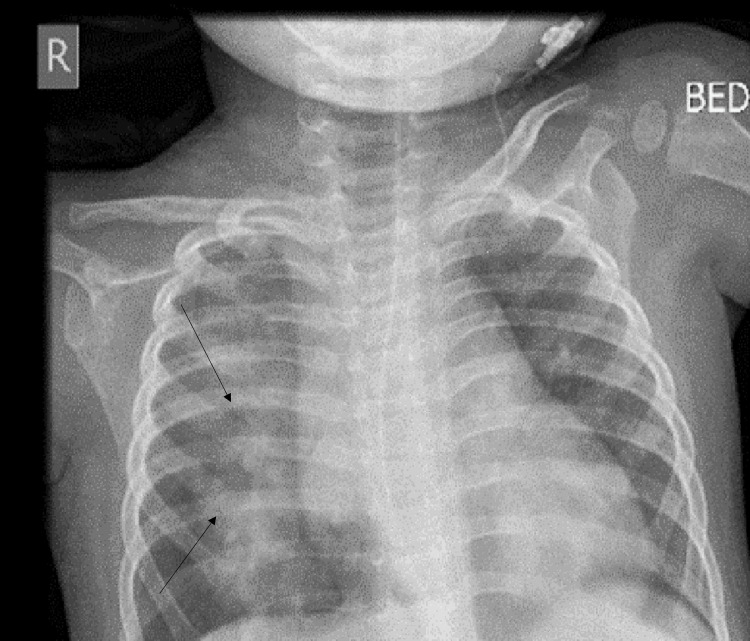
Confluent infiltrates in the right lung field.

**Figure 3:**
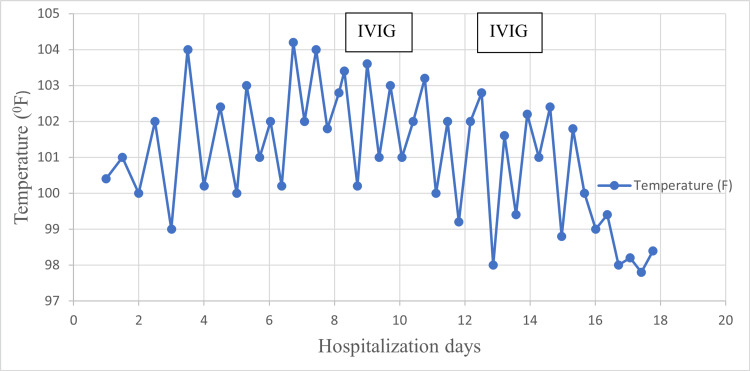
Temperature chart. IVIG: Intravenous immunoglobulins.

Treatment was commenced with a heated humidified high-flow nasal cannula (HHHFNC) at 18 l/min with fraction of inspired oxygen (FiO2) at 25% and saturation level at 96% on HHHFNC. Total leucocyte count (TLC) was 10,500/cmm (neutrophil 29%, lymphocyte 57%, monocyte 13%, basophil 1%), C-reactive protein (CRP) was 0.4 mg/dL, and international normalized ratio (INR) was 1.13. He had mild thrombocytopenia (platelet count 1.2 x105/cmm) and normocytic normochromic anemia (hemoglobin 9.9 gm/dL, mean corpuscular volume (MCV) 80 fL, mean corpuscular hemoglobin (MCH) 24 pg/cell). He was started on ceftriaxone and hypertonic saline nebulization and his AEDs were continued at his usual doses. His oxygen requirement gradually increased, and FiO2 was increased to 45% to maintain a saturation of 92-95%. On day five of admission, a chest X-ray showed infiltrates in the right lung field suggestive of pneumonia (Figure 2). He continued to experience fever spikes (Figure 3). Additionally, he developed loose motion on day five of admission. 

HHHFNC was maintained at 18 L/min with 45% oxygen with the antibiotics upgraded to a second line (cefoperazone/sulbactam) and azithromycin added. HAdV was detected in the reverse transcriptase-polymerase chain reaction (RT-PCR) of the nasopharyngeal swab. As fever spikes persisted, he was further investigated (Table 1).On day seven of hospitalization, TLC was 5,200/cmm, platelet count and hemoglobin (Hb) had dropped further, and inflammatory markers had gone up (CRP 5.65 mg/dL, serum ferritin 826 ng/mL). There was evidence of coagulopathy without obvious bleeding (INR 1.8), serum fibrinogen and triglyceride were 282 mg/dL (200-400) and 275 mg/dL (45-150), respectively, and serum albumin and serum sodium were 2.02 gm/dL and 128 mmol/L, respectively. 

**Table 1:** Serial blood investigations. Hb: Hemoglobin, TLC: Total leucocyte count, CRP: C-reactive protein.

Days of hospitalization	Day 1	Day 7	Day 9	Day 10	Day 11	Day 13	Day 15	Day 17
Parameters								
Hb (gm/dL)	9.9	7.0	10.6	10.0	8.8	8.5	8.1	7.1
TLC (/cmm)	10,500	5,200	3,600	8,800	12,200	12,400	15,100	18,900
% Neutrophil	29	64	38	31	41	29	28	35
% Lymphocyte	57	26	53	51	51	59	44	50
% Monocyte	13	10	7	18	7	10	25	11
% Eosinophil	0	0	2	0	0	02	2	2
% Basophil	1	0	0	0	1	0	1	2
Platelet (x10^5^/cmm)	1.29	0.74	0.46	0.38	1.15	3.49	11.26	16.81
CRP (mg/dL)	0.4	5.65					1.02	

He was transfused 15mL/kg of packed RBC on day seven of admission. Because of the patient's unrelenting fever (101-104^0^F), an absence of a clear microbial etiology (sterile blood and urine cultures on several occasions, ruling out malaria, dengue, enteric fever, and scrub typhus), elevated inflammatory markers, multisystem involvement (diarrhea, coagulopathy), and an epidemiologic link to SARS-CoV-2 infection with evidence of a prior SARS-CoV-2 infection (SARS-CoV-2 IgG 10.11, AU/ml, ≥ 10 considered reactive), MIS-C was diagnosed. He had no prior history of contact or exposure to SARS-CoV-2 infection, and there was no SARS-CoV-2 outbreak at the time in the community. Intravenous immunoglobulins (IVIG) was started on day eight of hospitalization and 2 gm/kg of IVIG was administered over the course of 48 hours. Due to the RT-PCR's HAdV positivity, steroids were not administered. Moreover, due to thrombocytopenia, aspirin could not be administered initially. He received a second dose of IVIG on day 13 due to his fever still being present after 48 hours of IVIG treatment. On day 15, within 24 hours of the last IVIG dose, defervescence took place. He remained afebrile since then. His platelet count started to rise and developed thrombocytosis by the end of the second week of admission (Table 1). Furthermore, he was found to be iron deficient (serum iron 18.5 mcg/dL, normal range 45-160). Echocardiography was suggestive of mild left main coronary arterial (LMCA) dilatation; 1 mm, z score -2.86. The child remained hemodynamically stable throughout his stay and didn’t require vasoactive drugs. He was weaned off HHHFNC on day 11 to low-flow oxygen support and was off oxygen support by day 14. He was discharged on day 17, on an anti-platelet dose of aspirin and iron supplement.

## Discussion

MIS-C is a rare complication of SARS-CoV-2 infection in children. We present a case of a male infant with hyperinflammatory syndrome with multiorgan involvement (diarrhea and coagulopathy) and vasculitis that was demonstrated by mild LMCA dilatation resembling Kawasaki disease. Moreover, the patient met the criteria of MIS-C. He did, however, have evidence of an active HAdV infection as established by a positive RT-PCR test for HAdV and evidence of prior exposure to SARS-CoV-2 indicated by positive SARS-CoV-2 serology.

The peak of coronavirus disease 2019 (COVID-19) cases in communities is followed by a lag of several weeks and then by an upsurge of MIS-C cases. This several-week lag matches with the period required for the acquired immunity indicating that MIS-C may be a postinfectious consequence of the virus rather than an acute infection. However, some children did have positive RT-PCR testing for SARS-CoV-2 infection indicating active infection at the time of hyperinflammation manifestation. In an early study from the United States, among the 539 children diagnosed with MIS-C, there were 437 children in whom both RT-PCR and serology were performed; 45% had positive serology with negative RT-PCR for SARS-CoV-2, 31% were positive on both tests, and 5% were RT-PCR positive and antibody negative [5].

Our case is distinctive in that, despite the presence of SARS-CoV-2 IgG antibodies (just above the cut-off), an RT-PCR test revealed positivity for HAdV and negativity for COVID-19. It is noteworthy to highlight that he had no prior history of exposure to or contact with SARS-CoV-2 infection and there was also no SARS-CoV-2 outbreak at the time that would have exposed him to it and given him an asymptomatic infection. The exaggerated immune response in the patient may have been brought about by an abnormal immune response to a past infection with SARS-CoV-2 or the HAdV infection may have triggered the hyperinflammatory condition.

Riphagen and colleagues were the first to report a series of eight children seen at a tertiary center in Southeast England during the COVID-19 pandemic with a hyperinflammatory syndrome and multiorgan involvement [4]. It is interesting to note that isolates of HAdV and enterovirus were discovered in one of the children.

The median age of MIS-C cases, which mostly affect children and adolescents, is nine years [5,6]. The patient in the current case presented in late infancy, which is an unusual age for the presentation of MIS-C. Also, this is the time when peak incidence of HAdV is observed, highlighting the possibility of HAdV-induced hyperinflammatory syndrome. Nevertheless, younger children do manifest MIS-C, and several neonatal cases and case series of neonatal MIS-C have also been reported [7,8].

## Conclusions

HAdV infection in immune-competent children is typically self-limiting and resolves with supportive care alone. However, HAdV infections have been associated with systemic inflammatory syndrome, notwithstanding its low incidence. A high index of suspicion in an infant with an HAdV infection who has a persistent fever, multiple system involvement, and elevated inflammatory markers, can prompt an early diagnosis of a hyperinflammatory syndrome that resembles MIS-C and Kawasaki disease, and it can be managed similarly to these conditions. This is particularly relevant when there hasn't been a recent documented COVID-19 outbreak in the community.
